# How Can the Microbiome Induce Carcinogenesis and Modulate Drug Resistance in Cancer Therapy?

**DOI:** 10.3390/ijms241411855

**Published:** 2023-07-24

**Authors:** Inês Mendes, Nuno Vale

**Affiliations:** 1 OncoPharma Research Group, Center for Health Technology and Services Research (CINTESIS), Rua Doutor Plácido da Costa, 4200-450 Porto, Portugal; inesmendes_2000@hotmail.com; 2 CINTESIS@RISE, Faculty of Medicine, University of Porto, Alameda Professor Hernâni Monteiro, 4200-319 Porto, Portugal; 3 School of Life and Environmental Sciences, University of Trás-os-Montes and Alto Douro (UTAD), Edifício de Geociências, 5000-801 Vila Real, Portugal; 4 Department of Community Medicine, Information and Health Decision Sciences (MEDCIDS), Faculty of Medicine, University of Porto, Rua Doutor Plácido da Costa, 4200-450 Porto, Portugal

**Keywords:** carcinogenesis, cancer hallmarks, human microbiome, altered microbiome, gut microbiome, drug resistance, genetic instability, epigenetic modifications, personalized medicine

## Abstract

Over the years, cancer has been affecting the lives of many people globally and it has become one of the most studied diseases. Despite the efforts to understand the cell mechanisms behind this complex disease, not every patient seems to respond to targeted therapies or immunotherapies. Drug resistance in cancer is one of the limiting factors contributing to unsuccessful therapies; therefore, understanding how cancer cells acquire this resistance is essential to help cure individuals affected by cancer. Recently, the altered microbiome was observed to be an important hallmark of cancer and therefore it represents a promising topic of cancer research. Our review aims to provide a global perspective of some cancer hallmarks, for instance how genetic and epigenetic modifications may be caused by an altered human microbiome. We also provide information on how an altered human microbiome can lead to cancer development as well as how the microbiome can influence drug resistance and ultimately targeted therapies. This may be useful to develop alternatives for cancer treatment, i.e., future personalized medicine that can help in cases where traditional cancer treatment is unsuccessful.

## 1. Introduction

Through the course of history, there has been an enormous effort from the scientific community to investigate the mechanisms that lead to the transformation of a normal cell into cancer. Therefore, different theories have emerged ranging from cancer seen as a malediction to the comprehension at the level of single-cell heterogeneity, suggesting that there exists countless molecular challenges to overcome even between a single sub-type of cancer. Because of the development of different fields such as cancer genetics and biology, this disease is becoming an enormous challenge to elucidate. It is important to understand that usually tumor cells do not create new mechanisms; instead, they start controlling molecular and cellular pathways that already exist to escape protective systems which are in place to avoid the formation of a tumor [1].

The technological progress of the “omics” such as genomics, proteomics, epigenomics, pharmacogenomics, and metabolomics enabled the improvement of the diagnosis, prognosis, and treatment of cancer. Due to this development, today, precision medicine is a clinical reality [2]. In recent decades, the fast development of new therapeutic strategies has significantly helped to reduce mortality in patients with cancer [3]. However, drug resistance is one of the most challenging topics to investigate in cancer research because it still represents a huge obstacle to treat this disease. So, decoding the mechanisms of drug resistance is essential to fully comprehend the multifactorial pathways involved in cancer, and this may provide information to develop specific targeted treatments [4].

Theodor Boveri observed that tumor cells had abnormal chromatin structures and by that observation he gave the first insights for the role of epigenetics in cancer [5]. The first author to coin the term ‘epigenetics’ was Conrad Waddington and he defined it by being the ‘the branch of biology which studies the causal interactions between genes and their products which bring the phenotype into being’ [6]. Vogelstein and Feinberg tried to determine the mechanisms behind the higher frequency of mutations existing in tumor cells—in comparison to normal tissue, the tumor tissue had lost DNA methylation to a considerable degree, suggesting that hypomethylation of CpG islands may result in oncogene activation in cancer [7]. So, this work enabled understanding how frequent hypomethylation is in tumor genomes [1]. Holliday enhanced the concept of epigenetics as heritable modifications that occur in gene expression without changing the DNA sequence; in other words, modifying the phenotype without changing the genotype [8].

It is becoming clear that epigenetics plays a key role in carcinogenesis by altering the gene expression and there are various situations in which this occurs, for example, the hypermethylation of tumor-suppressor genes in retinoblastoma [9], as well as epigenetic silencing of microRNAs [10]. It is also known that the epigenetic mechanisms ensure the maintenance of genomic integrity and faithful genome replication in the cell cycle. Transposable elements (TE) are extremely repetitive sequences of DNA that are present in the human genome, they possess their own regulatory sequence, and this allows autonomous expression and capacity to modify the expression of neighboring genes. Because TE activity has a high susceptibility to disturbing genomic integrity, these are commonly silenced genes by epigenetic mechanisms; however, this regulation is lost in cancer [11]. Recently, epigenetic dysregulation was proposed to be a pivotal hallmark of cancer because it is a unique feature that is found within cancer cells (there are epigenetic fingerprints on tumor cells), so it is an active and functional ability that these cells have that confer their chronic nature. This completely changes the view of epigenetic dysregulation seen as a simple by-stander and finally recognizes its active role in tumorigenesis [1].

The microbiology field is used to characterize microorganisms, for example, viruses, bacteria, fungi, archaea, and protozoa, and associates them with the pathogenesis of human diseases. There is an interactive ecosystem among the human microbiome that includes various microorganisms, and they continuously interact with the environment as well as the host, particularly the immune system [12,13]. Recent evidence shows the importance of endogenous and exogenous microorganisms in the pathogenesis of different neoplasms [14,15,16] in addition to non-neoplastic diseases [17,18]. Therefore, among the microorganisms with established or probable carcinogenic effects are *Helicobacter pylori* and Epstein–Barr virus for gastric carcinoma [19]; hepatitis B and C viruses (HBV and HCV) for hepatocellular carcinoma (HCC) [20]; human herpesvirus-8 for Kaposi’s sarcoma [21]; human immunodeficiency virus for Kaposi’s sarcoma, aggressive B-cell non-Hodgkin lymphoma, and cervical carcinoma [21]; HPV for uterine cervical, anal, and oropharyngeal carcinomas [22,23]; human T-lymphotropic virus type 1 for adult T-cell leukemia/lymphoma [24]; *Fusobacterium nucleatum* for colorectal carcinoma [25]. Moreover, it is possible that the tumors that result from carriers of these pathogenic microorganisms have different molecular pathological characteristics, in comparison with tumors that arise in non-carriers [26]. Several studies have shown that modifications of the microbial ecosystem are also important in the pathogenesis of numerous neoplasms [27,28]. Pathogens may promote cancer development using distinct genetic processes [29]. One of the most important among described biologic carcinogenic agents for humans is *Helicobacter pylori* (*H. pylori*), which has been shown to lead to inflammation as well as gastric cancer [29,30,31]. It is described that *H. pylori* is not only capable of triggering cancer, but that *H. pylori* combined with different microorganisms promotes violent gastrointestinal intraepithelial neoplasia [32].

The present review was conducted to collect the most recent findings about the mechanisms by which an altered microbiome induces genetics and epigenetics modifications on the human body involved in carcinogenesis as well as mechanisms such as drug resistance moving towards personalized medicine. The PubMed and B-ON search was performed, and we used the keywords “Microbiome” and “Cancer” together or along with one more keywords, for example, “Epigenetic modifications”, “drug resistance”, or “genetic instability”. Full-text articles published in English in 2015 or later were considered. In addition, for further elucidation and a deeper understanding, we also considered different older articles as well. Abstracts, case reports, editorials, commentaries, or manuscripts published in languages apart from English were not considered for this review.

## 2. Drug Resistance in Cancer

Elucidating the pathways of resistance to treatment in human cancer cells has grown into a multifaceted constraining factor to obtain adequate cures in cancer patients. Apart from genetic and epigenetic modifications, increased DNA damage repair activity, uncontrolled cell death, overexpression of transmembrane transporters, as well as complex relations that occur inside the tumor microenvironment, additional ways of cancer therapy resistance have been suggested recently. Increasing evidence from preclinical as well as clinical studies have been focusing on the essential role of microbiota not only in cancer initiation and progression but, equally importantly, in the success of anticancer treatments, usually chemo- and immunotherapy [33].

### Cancer and Infectious Diseases

The issue regarding drug resistance is remarkably similar to the area of infectious disease because it is also challenged by highly proliferating intrinsic or extrinsic aggressors. So, comparing what happened with antimicrobial therapy, the first chemotherapeutics (such as nitrogen mustard [34] and aminopterin [35]) used in patients showed initial success; however, this initial excitement vanished because the results started showing that even though tumors went into remission rapidly, they acquired resistance, and the result was the disease relapsed. To overcome the resistance to single-agent chemotherapy, another approach was performed; without surprise it was taken from the rulebook of antimicrobial therapy [36] and so it combined the administration of agents with non-overlapping mechanisms of action, or polychemotherapy. The results showed that this approach worked particularly well in several forms of lymphoma, breast cancer as well as testicular cancer [37,38,39].

In cancer therapy, polychemotherapy has become a new paradigm and enabled the development of increasingly complex regimens. Additionally, several different methods to dose intensity [40], as well as shorter-interval administrations of chemotherapy [41,42] or even higher doses of chemotherapy [41] with growth factor support to avoid continued bone marrow suppression, revealed an improvement of success of these therapies by avoiding early regrowth of tumors. At the turn of the century, approximately 50 years after its introduction, the advances accomplished with polychemotherapy had plateaued. It was observed that surgery, radiotherapy as well as polychemotherapy were certainly insufficient to treat various tumor types. Therefore, new therapeutic strategies started to be developed aiming to target the key enabling characteristics as well as the obtained abilities that allow cancer cells to transform normal cells and tissues into malignancies. The emergence of therapies that disturbed these hallmark characteristics [43,44] in which targeted therapies are included was a step forward towards cancer treatment. In fact, the elucidation of the biological characteristics of cancer has led to highly efficient therapies against tyrosine kinases, nuclear receptors, as well as other molecular targets. The first successful results of estrogen receptor (ER) and androgen receptor (AR) antagonists, in addition to BCR-ABL, HER2 and EGFR inhibitors, resulted in an enormous attempt to develop agents that target oncogenes as well as critical cellular weaknesses. In recent years, there was a new advance in oncological therapy using immunological methods to identify and attack cancer. There are monoclonal antibodies such as anti-CTLA4 [45] and anti-PD-1/PD-L1 [46] that impair negative regulators, or checkpoints, of the adaptive immune system, leading to significant antitumor activity—and even cures—in different tumor types [47].

Previously, using the typical chemotherapy treatment, some resistance was observed, and this is also verified in targeted and immunological therapies, so it continues to be highly frequent. This is where the correlations regarding cancer and infectious diseases can differ: combination therapy usually results, for instance, in disease becoming unnoticeable in HIV or treated in tuberculosis; however, in metastatic cancers, this ends up being the exception instead of the rule [48]. As expected, cancer is a more complex biological disease [49].

## 3. The Human Microbiome

Microbes begin to colonize our body at early stages of fetal development, particularly in the 2nd trimester, in which reduced levels of microbial signals may be identified in the fetal gut, skin, placenta, as well as lung tissue [50]. Nevertheless, the first main colonization episode in early life occurs at birth, in which the form of childbirth molds the neonate microbiome constitution to be similar to either a vaginal or skin microbiome [51]. Afterwards, our microbiomes are molded by external influences including diet, lifestyle, as well as surrounding biological diversity [52]. Our microbiome constitution changes with age. In early newborn life, breastfeeding allows the vertical transmission (mother to child) of bacteria. Consequently, neonates reveal a microbiome constitution similar to their mother’s milk [53].

There are trillions of microbes inhabiting the human bodies and a co-evolution exists between humans and microbes, so they created mechanisms to be admirably adapted to the host physiology that is in constant change [54]. Despite most of these microbes reside inside our gut, different groups of microbes are also present in most body parts, in which the brain may be included; however, recent evidence is not conclusive [55]. The microbiome of every body part has different aspects in terms of population dynamics as well as the variety of microbial species [56]. This site-specific variety and dynamics may be considered as a health indicator [57]. Studies have made it possible to establish that the microbial environment in the human body is essential regarding health preservation by their association with the nutrient absorption, the immune system, as well as different metabolic pathways. Therefore, in a symbiotic state, host–microbe relations counteract invading pathogens and avoid tumor formation [58].

### 3.1. Altered Human Microbiome and Carcinogenesis

It has been emerging and expanding the elucidation of the variety as well as the differences in the abundance of microorganisms, together named the microbiota. They make symbiotic associations with the barrier tissues of the human body that are found to be in direct contact with exterior environment—the epidermis as well as the internal mucosa, particularly the gastrointestinal tract, in addition to the lung, the breast, as well as the urogenital system. There is increasing comprehension that the ecosystems established by inhabitant bacteria as well as fungi—the microbiomes—have a significant effect on the organism’s health but also disease [59], a recognition promoted by the possibility to analyze the populations of microbial species through next-generation sequencing in addition to bioinformatic tools. In fact, for cancer, the data are progressively becoming consistent that polymorphic differences in the microbiomes among individuals within a population may have a remarkable influence on cancer phenotypes [60,61].

Different association investigations in humans as well as investigational manipulation in mouse models of cancer are showing that specific microorganisms, mostly but not only bacteria, may have either protective or harmful influences on carcinogenesis, malignant evolution, as well as treatment reaction—as may the total complexity and composition of a tissue microbiome in general. In fact, although the intestinal microbiome has led the way, many tissues as well as organs have their respective microbiomes that reveal unique features regarding population dynamics as well as variety of microbial species and subspecies. Nevertheless, it is still not elucidated if the microbiome is a discrete empowering feature that has significant positive and negative influences on the gain of hallmark capacities for cancer [59].

**Figure 1:**
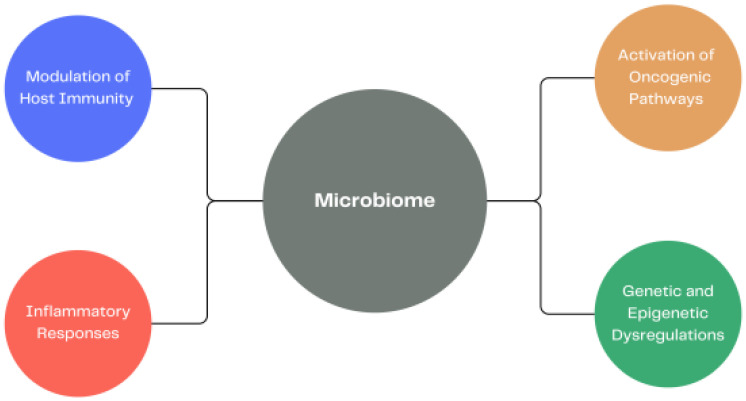
Different consequences of an altered human microbiome that may lead to cancer.

Microbiota is currently starting to be identified as an essential player in carcinogenesis as well as the interactions between microbes are higher than it was previously predicted [62]. As arising tumor elements, intratumoral bacteria were discovered in various solid tumors and different studies have shown that various cancer subtypes have different microbial constitutions. Other mechanistic research observed that intratumoral bacteria can lead to cancer initiation and development by inducing DNA damage, epigenetic alterations, inflammatory responses, modulation of host immunity as well as the activation of oncogenes or oncogenic pathways (Figure 1) [63]. It was observed that the causative agents of cancer were H. pylori, human papilloma virus, hepatitis B virus (HBV) and hepatitis C virus (HCV); however, it is important to mention that the composition of the human microbiome consists in 10–100 trillion microbial partners, most of them are not identified [64]. This fact shows that we should not underestimate the role the microbiome plays in cancer and that more research in this field is required.

In studies designed to analyze the impact that pathogenic bacteria have in promoting epigenetic dysregulation it was observed that they play a significant role in the dysregulation of the epigenetic machinery of their target human cells. It was also important to notice that they did not only promote this directly, but these bacteria produce toxins and surface proteins targeting the host human cell membrane and they can also synthesize effector proteins entering the host cell nucleus. It was observed that all these products usually have consequences such as changes in the host human cells DNA methylation patterns, histone alterations or in other words modifications of the “histone code”. Therefore, the alterations in the host human cells epigenotype and in the gene expression pattern can interfere with the activity of the antibacterial immune response and produce a propitious environment for bacterial colonization, growth, or spread [65].

The production of inflammatory cytokines and other inflammatory mediators is promoted by epigenetic dysregulation mediated by bacterial products disturbing the epigenotype of their target human cells. The indirect epigenetic modifications in addition to the direct interference with the epigenetic machinery of the host human cells is a promising topic in the cancer research field since they can trigger the initiation and progression of malignant tumors related with different bacterial infections [65]. Different studies have shown that bacteria included in such wide phyla as Firmicutes, Actinobacteria, Bacteroidetes, Chlamydiae, Fusobacteria, and Proteobacteria end up encoding proteins or processing cell wall components that can interact with the epigenetic machinery of host human cells [66,67,68].

### 3.2. The Importance of the Gut Microbiome

#### Varied Modulatory Influences of the Gut Microbiome

It has been described that the intestinal microbiome is remarkably essential regarding the role of the large intestine (the colon) in degrading as well as importing nutrients into the body in the context of metabolic homeostasis, and that disturbances in the microbial populations—dysbiosis—in the colon may provoke a range of different physiologic diseases [59]. Therefore, there has been the intuition that the predisposition, progress, as well as pathogenesis of colon cancer are affected by the intestinal microbiome. Recently, credible functional investigations that involved fecal transplants from colon tumor-bearing patients but also mice into recipient mice susceptible to being affected by colon cancer have recognized that there are both cancer-protective as well as tumor-promoting microbiomes, encompassing specific bacterial species that may modulate the prevalence as well as pathogenesis of colon tumors [69].

The processes by which microbiota confer these modulatory functions are even now being clarified; however, two common outcomes are becoming progressively well recognized for tumor-promoting microbiomes and, in several situations, for particular tumor-inducing bacterial species. The primary consequence is mutagenesis of the colonic epithelium, resulting from the production of bacterial toxins as well as different molecules that impair DNA directly, or disturb the mechanisms that preserve genomic integrity, or stress cells using other methods that indirectly damage the fidelity of DNA replication and repair. One example is *E. coli* carrying the PKS locus, which evidently mutagenizes the human genome and is involved in the transmission of hallmark empowering mutations [70]. In addition, bacteria have been described to bind to the surface of colonic epithelial cells and generate ligand mimetics that promote epithelial proliferation, promoting in neoplastic cells the hallmark ability for proliferative signaling [60]. Additional process in which particular bacterial species lead to tumorigenesis include butyrate-producing bacteria, the incidence of which is increased in individuals affected by colorectal cancer [71].

The generation of the metabolite butyrate has complicated physiological consequences, such as the generation of epithelial cells and also of fibroblastic cells. It was observed that a mouse model of colon carcinogenesis inhabited by butyrate-producing bacteria revealed additional tumor development, and when compared to mice that lacked these bacteria; the association between butyrate-induced senescence of epithelial cells and increased colon tumorigenesis has been proved with the utilization of a senolytic drug that destroys senescent cells, and which have damaged tumor development [71]. Furthermore, butyrate produced by bacteria has pleiotropic as well as paradoxical influences on differentiated cells versus undifferentiated (stem) cells in the colonic epithelium under circumstances in which the gut barrier is disturbed (dysbiosis) and the bacteria are invasive, impacting, for instance, cellular energetics as well as metabolism, histone modification, cell-cycle progression, but also (tumor-promoting) innate immune inflammation which is immunosuppressive of adaptive immune responses [72].

In addition to causal associations to colon cancer and melanoma, the intestinal microbiome’s evident capacity to stimulate the expression of immunomodulatory chemokines as well as cytokines that are released in the systemic circulation is also clearly able to influence cancer pathogenesis and treatment reaction in different organs of the body [15,73]. A revealing case includes the progression of cholangiocarcinomas in the liver: intestinal dysbiosis enables the entry and transport of bacteria as well as bacterial products through the portal vein to the liver, in which TLR4 is expressed on hepatocytes is triggered to stimulate the expression of the chemokine CXCL1, that recruits CXCR2- expressing granulocytic myeloid cells (gMDSC) which functions to suppress natural killer cells so as to evade immune destruction, and possible confer other hallmark abilities. Thereby, the intestinal microbiome is unequivocally involved as an enabling feature that may instead help or confer protection against various types of cancer [74,75].

## 4. Examples of Microbes and Their Association with Several Cancer Types

Every tissue and organ in contact, directly or indirectly, to the external environment is also a repository for commensal microorganisms [76]. Contrasting with the gut, where the symbiotic function of the microbiome in metabolism is clearly identified, the normal and pathogenic functions of microbiota residing in these distinct sites are still being elucidated. Apparently, there are organ/tissue-specific distinctions in the composition of the corresponding microbiomes in homeostasis, aging, as well as cancer [76,77]. Furthermore, association investigations are reporting more and more data regarding local tumor-antagonizing/protective versus tumor-promoting tissue microbiomes, in the same way as the intestinal microbiome may modulate predisposition and pathogenesis to human cancers developing in their related organs [78,79,80,81].

### 4.1. Colorectal Cancer

Recently, various authors have described the effect of the gut microbiome in the development of colorectal cancer (CRC) [82]. Because the CRC occurrence is currently being described in young adults, and it is also reported as the second most common cause of death throughout the world, precise knowledge regarding the role that gut microbes plays in the progress of colorectal cancer is becoming urgent to elucidate. So, in this review, we describe the current knowledge regarding the effect of gut microbes on the genetics as well as epigenetics of colorectal cancer [82]. Imbalances in the gut flora are related to colorectal cancer, curiously numerous investigations have shown that *Fusobacterium* is associated with colorectal tumor [83,84,85,86]. However, the incidence of distinct bacteria such as *Leptotrichia*, *Prevotella*, *Gemella*, *Porphyromonas*, *Peptostreptococcus*, *Parvimonas*, *Campylobacter* are linked with colorectal cancer [87,88].

Zackular et al. (2014) have described that when feces from people affected by cancer are analyzed, they reveal to carry distinct bacteria, in addition to an excess of the traditional mouth bacteria, *Fusobacterium* or *Porphyromonas* [89]. In addition, Zeller et al. (2014) described the relations occurring between the gut microbiota and cancer, it two *Fusobacterium* species were observed, specifically *Peptostreptococcus stomatis* as well as *Porphyromonas asaccharolytica*, and they revealed to be increased in colorectal cancer patients compared to the healthy individuals [90]. Moreover, with new technology development, the meta-transcriptome analyses provided data that exposed a significant increment and simultaneous presence of *Leptotrichia* genera and *Fusobacterium.* Furthermore, *Campylobacter* in CRC tumors that are Gram-negative bacteria exist in the oral cavity; however, both *Campylobacter* as well as *Fusobacterium* are inherently distinct from their oral counterparts [88]. In addition, Burns et al. discovered *Providencia* within the tumor microenvironment [91]. Further, it was determined that initiation of inflammatory reactions through commensal bacteria contributes to tumor development as well as growth [92]. *Enterococcus faecalis* generates DNA-damaging superoxide radicals as well as genotoxins that are drivers that may promote CRC development [93].

Apart from these driver bacteria, the passenger microorganisms that comprise bacteria as well as several viruses additionally promote the development of cancer. As stated in the CRC ‘driver-passenger’ model, symbiotic ‘driver’ bacteria contribute to tissue tumor formation through cell DNA damage, and colorectal tumorigenesis is then mediated by alterations in the intestinal microenvironment, that contributes to the growth of “passengers” opportunistic pathogens such as *Fusobacterium* spp., *Streptococcus bovis*, and *Roseburia* spp. [94]. The Human Polyoma Viruses (HPyVs) are another example that can also be listed among the “passenger” players. HPyVs infections are usually asymptomatic, regardless of their described transformative abilities, with an age profile indicating an elevated incidence of early-age infections and that it remains throughout life [95,96]. It is important to note that gut bacterial as well as viral dysbiosis is a main reason for modified host immune response in both CRC and obese patients, resulting in a lasting inflammatory state [97].

### 4.2. Breast Cancer

All over the world, breast cancer (BC) in men is a rare disease; however, it continues to affect the lives of millions of women and it is the most frequent type of cancer in terms of incidence among women [98]. Breast cancer includes a heterogeneous group of neoplasms with various morphologies, molecular phenotypes, therapy reactions, probabilities of recurrence and general survival [99]. It is not a single disease; instead, there are various distinct cancers and every single one of them influences the breast. Molecular subtyping considering the presence or absence of cell surface receptors, for instance ER, PR and Her2 provides the information required for the right treatment strategy. In situations where it is observed that there is an absence in patients of all three receptors or markers, or triple-negative breast cancer (TNBC), they have various adverse outcomes [100]. The reason this occurs is mostly because of the non-existence of targeted therapies and it is another example of how important it is to study cancer hallmarks aiming to understand how targeted therapies can be developed.

Bacterial inhabitants of the human body have the possibility to interfere in different phases of cancer initiation, development, as well as therapy. These bacteria can be distal to the primary tumor, for example gut microbiota, or local to the tissue, previous or posterior tumor growth [101]. Different investigations have shown that the gut microbiome of patients affected by breast cancer changed compared to healthy matched controls [102]. Considering the importance of microbial dysbiosis in chronic inflammation, inflammation-mediated carcinogenesis processes, and immune evasion, it was expected that certain microbes contributed to the growth of some types of cancers. As it was mentioned before, such relationships have been observed with the role of *H. pylori* in gastric cancer as well as *Fusobacterium* in colorectal cancers [99,103,104]. Nevertheless, there is not extensive knowledge about the relations that exist between the microbiome and breast cancer [105]; therefore, it may be an interesting topic for future research.

**Figure 2:**
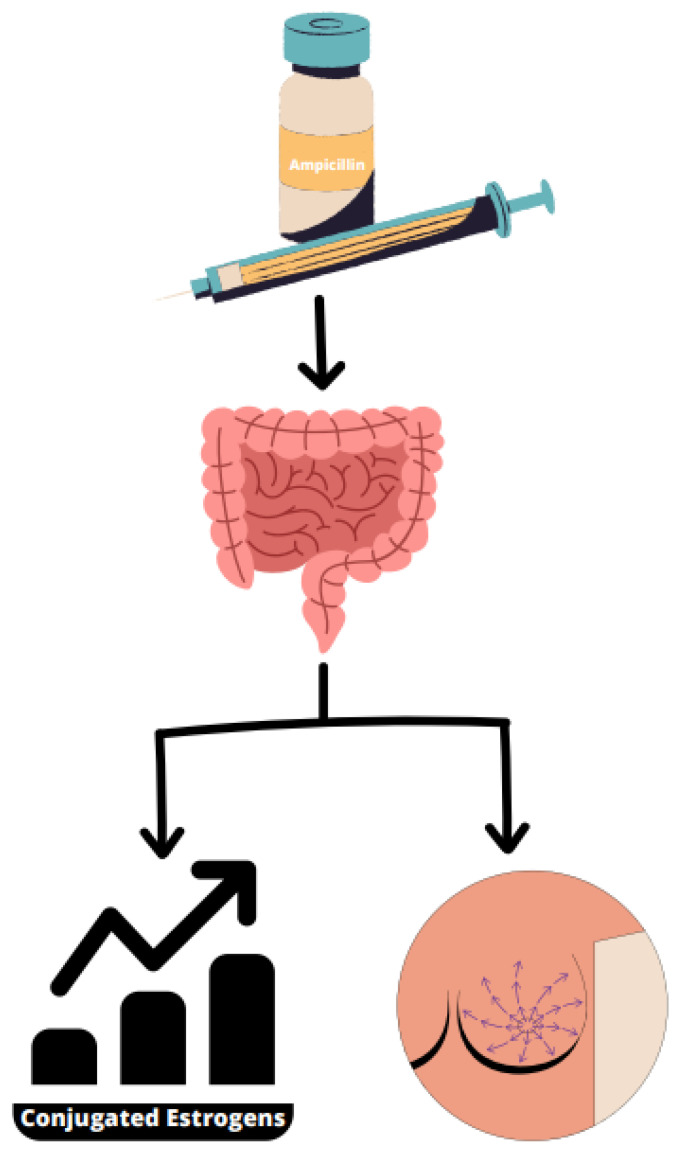
The influence of the gut microbiota in estrogen metabolism using ampicillin treatment on patients showing that it may have impact on breast cancer because of the increase in fecal excretion of conjugated estrogens.

The influence of the microbiome environment on the metabolism of estrogen is becoming increasingly evident, where there exists a solid association with breast carcinogenesis. This fact was observed in one investigation in which patients that obtained ampicillin treatment had higher fecal excretion of conjugated estrogens, highlighting the active impact of the gut microbiota in estrogen metabolism (Figure 2) [106]. This indicates that gut microbes can be implicated in the metabolism of estrogen; therefore, altering one’s microbiome can have several consequences on breast cancer pathogenesis. Additionally, sex hormones may also influence the gut microbiome constitution [107]. One case–control research revealed that the fecal microbiota of postmenopausal breast cancer patients showed reduced variety as well as general distinct constitution in comparison with matched controls [102]. Another investigation described identical results with an increase in *Methylobacterium radiotolerans* in breast tumor tissue compared to *Sphingomonas yanoikuyae* in corresponding healthy tissues. Essentially, quantification of total bacterial DNA load exhibited an inverse association among bacterial load and breast cancer disease stage. Stage 1 patients carried the highest copy numbers of bacterial DNA in comparison with both stage 2 and 3 patients. This difference in bacterial load was also related to lower expression of antibacterial response genes between advanced stage breast cancer patients. These results indicate that dysbiosis can play a role in breast cancer tumor development, in which a lower or modified bacterial constitution may promote downstream abnormal immune system functioning allowing tumorigenesis. In addition, these results indicate that bacterial load may be used as a biomarker for diagnosis as well as staging, therefore requiring further research [107].

The breast tissue and milk were considered sterile; however, they are currently recognized to incorporate a varied as well as specific microbial community [108,109]. A study that compared the microbial constitution of nipple aspirate fluid in women that possess history of breast cancer versus normal controls exhibited a comparatively increased prevalence of the genus *Alistipes* as well as reduced prevalence of a genus from the *Sphingomonadaceae* family. Further investigations show that the microbiome of breast skin swabs as well as breast tissue obtained from patients with breast cancer in comparison with health controls is increased in specific microbes, such as *Fusobacterium*, *Atopobium*, *Gluconacetobacter*, *Hydrogenophaga*, *Bacillus*, *Enterobacteriaceae*, *Staphylococcus*, *Comamonadaceae*, as well as *Bacteroidetes* [110,111,112].

Predominantly, research on microbiome and its impact on breast diseases has highlighted the comprehension of the associations regarding invasive cancers; nevertheless, non-malignant breast diseases are usual and may adversely influence the quality of living which includes a higher probability of cancer [113]. This kind of non-malignant breast diseases consist of Atypical Ductal Hyperplasia (ADH), Ductal Carcinoma In Situ (DCIS), as well as mastitis/breast abscesses. Although specific microorganisms, most remarkably *S. aureus*, have for a long time been associated as causative in mastitis, recently a study revealed that milk obtained from mastitis patients exhibited microbiota disruptions such as reduced microbial variety with higher opportunistic pathogens and lower commensal organisms [114]. ADH as well as DCIS, represented by aberrant, neoplastic cell proliferation and perhaps promoting invasive breast cancer, have several recognized risk factors; however, their etiology is not widely known. Supplied data indicating that microbial dissimilarities in different tissues may be related with neoplastic non-malignant growth [115]; the doubt is whether the breast and gut microbiomes can affect non-malignant breast diseases like ADH as well as DCIS [105].

### 4.3. Gastric Cancer

#### Helicobacter Pylori and Gastric Carcinogenesis

*Helicobacter pylori* is the most frequent bacterium which means it is almost ubiquitous in humans; it colonizes the gastric epithelium of approximately 50% of people worldwide and has been co-evolving with humans in an interaction spanning 50,000 years [116,117]. *H. pylori* colonization offers protection against demyelinating diseases including tuberculosis [118], multiple sclerosis [119] and inflammatory bowel disease [120]. It was observed that in fact it has several beneficial roles; however, it is important to mention that it was the first bacterial carcinogen reported and it is linked to 90% of gastric cancers [121,122]. Due to the toxins that the bacteria produce occurs gastric oxidative stress and reactive aldehyde formation. Another important consequence is related to the production of cellular DNA and RNA damage and hypermethylation of DNA promoter genes. Moreover, *Helicobacter pylori* toxins may also provoke host inflammatory response, chronic mucosal inflammation, achlorhydria, synergistic interactions with other carcinogens, as well as making the antioxidant protection inefficient in the gastric mucosa [123].

*Helicobacter pylori* induces pathogenicity as well as the gastric carcinogenesis which appear to be related to various virulence factors that are vacuolating cytotoxin A (VacA), obviously depending on the expression of *vacuolating cytotoxin gene A* (*vacA*); *cytotoxin-associated gene* pathogenicity island (*cag*PAI); an oncoprotein (i.e., cytotoxin-associated gene A (CagA)), as well as adhesion proteins [124,125,126,127]. It is important to understand how this works because *CagA*-positive *H. pylori* contributes to the creation of genetic instability by disturbing the mitotic spindle checkpoint, leading to chromosomal instability [128] as well as epigenetic instability [129] and can ultimately lead to gastric carcinogenesis.

## 5. Influence of Intratumoral Microbiota

Pathologists have long established that bacteria may be discovered inside solid tumors, a finding that has now been demonstrated with advanced profiling tools. For instance, an investigation of 1526 tumors containing seven human cancer types (bone, brain, breast, lung, melanoma, ovary, as well as pancreas), every form was identified by a characteristic microbiome that was mostly located within cancer cells and immune cells, and inside every tumor type, changes in the tumor microbiome might be discovered as well as deduced to be linked to clinicopathologic characteristics [130]. Microbiota have also been discovered in genetically engineered de novo mouse models of lung and pancreas cancer, and its deprivation in germ-free mice and/or their abrogation with antibiotics may evidently damage tumorigenesis, functionally involving the tumor microbiome as a facilitator of tumor-promoting inflammation as well as malignant development [131,132].

Association investigations in human pancreatic ductal adenocarcinoma as well as functional exams through fecal transplants into tumor-bearing mice have recognized that changes in the tumor microbiome—and the corresponding intestinal microbiome—modulate immune phenotypes and survival [133]. A significant task for the future will be to expand these effects to different tumor types, as well as to define the possibly independent influences of composition and changes in the tumor microbiome to that of the intestine (as well as local tissue of origin) microbiome, possibly by recognizing certain microbial species that are essentially important in one site or another [75].

## 6. Epigenetics

Epigenetics comprehends heritable structural as well as biochemical changes in the chromatin with no alterations on the DNA sequence [134]. Epigenetic processes control different physiological as well as pathological mechanisms by regulations of important gene expressions through modifying the ease of access of epigenetic codes to the chromatin locally but also globally [135,136,137].

There are three fundamental epigenetic codes that have been admirably investigated consisting of DNA methylation, histone modifications as well as non-coding RNAs (ncRNAs). DNA methylation is the most significant epigenetic process that has been intensively studied. There are distinct DNA methylation alterations for example 5-methylcytosine (5 mC), N6-methyladenine (6 mA) as well as 4-methylcytosine (4 mC) [138,139]. Although 6 mA and 4 mC are usually discovered in prokaryotic genome, 5 mC is the most broadly dispersed methylation type in eukaryotes, but also the most investigated and comprehended DNA alteration pattern in general [140].

There are various methodologies typically used to assess the estimated or precise methylation contents of DNA. Bisulfite conversion is the basis for most of DNA methylation assays that converts cytosine to uracil in single-stranded DNA; however, it does not impact 5 mC [141]. Additional methods consist of digestion of genomic DNA using particular endonucleases with distinct methylation sensitivities aiming to obtain an approximate prediction of the totality of DNA methylation [142]. Because of these changes, DNA methylation status in particular loci or global contents may be assessed using various methodologies. It is important to notice that not every RNA transcript will ultimately lead to proteins, some of them have regulatory functions. ncRNAs are a group of RNA transcripts that do not encode proteins as mRNAs do [143]. ncRNAs have been recognized as by-products of protein transcription with reduced biological functions. Indeed, ncRNAs have been associated as essential epigenetic regulators that are actively involved in many physiological as well as pathological mechanisms [144].

The growing epigenetics field will possibly promote an increase in high-throughput sequencing technologies, which will create a potential possibility to decode the nature of the epigenome at the systematic level. Therefore, bioinformatic and biostatistics technologies/pipelines are crucial for processing of large volumes of datasets as well as providing helpful knowledge in this “omic” era [145]. The essential advance in the area of epigenetics encourages the progress of improved tools in support of finely designed technologies and mechanisms to identify, quantify, as well as visualize the dynamics of chromatin state [146].

### Non-Mutational Epigenetic Reprogramming

The empowering feature of genome (DNA) instability, as well as mutation is a key element of cancer development and pathogenesis. Currently, several international consortia are categorizing mutations around the genome of human cancer cells, therefore in virtually all types of human cancer, at distinct stages of malignant development, in which metastatic lesions are included, and through the progress of adaptive resistance to treatment. As a consequence, the current extensive comprehension that mutations in genes that organize, modulate, as well as preserve chromatin architecture, and thus as a whole regulate gene expression, are more and more being identified and functionally linked with cancer hallmarks [147,148,149]. There is, additionally, a situation to be presented for another evidently independent way of genome reprogramming that simply includes epigenetically regulated modifications in gene expression, which could be named “non-mutational epigenetic reprogramming”. In fact, the idea of mutation-less cancer progress and just epigenetic programming of hallmark cancer phenotypes appeared practically a decade ago [150] and is gradually more debated [147,151,152,153].

The notion of non-mutational epigenetic regulation of gene expression is clearly recognized as the main process mediating embryonic growth, differentiation, as well as organs development [154,155,156]. In the adult, for instance, long-term memory includes alterations in gene and histone modification, in chromatin structure, as well as in the induction of gene expression changes that are stably preserved in the course of time by positive and negative feedback loops [157,158]. Increasing data confirm the idea that analogous epigenetic modifications may support the gain of hallmark abilities through tumor progression and malignant development [75].

## 7. Epigenetic Regulation of CRC

The DNA methylation is the most investigated epigenetic modification, in CRC [159]. Abnormal DNA methylation is promoted in driver genes through CRC progress [160,161,162], in addition, histone alterations also influence CRC growth [163,164]. Circulating nucleosomes transport CRC related histone marks, for instance, H3K9me3, H4K20me3, as well as H3K27me3 [165]. Furthermore, the trimethylation of histones H3K4, H3K9, and H4K20 was linked to CRC survival and relapse [166].

It was observed several genes that become hypermethylated in CRC such as *APC*, *MGM2*, *RAAS F2A*, *RUNX3*, *HLTF*, *ALX4*, *SOX2*, *p14*, *p16*, *DLCK1*, *WIF1*, as well as *NDRG4* [167]. Curiously, the left side of colon reveals different features from the right side regarding the methylation level of distinct genes, microsatellite instability (MSI), types of mutations, as well as reaction to therapy. The designation CpG Island Methylator Phenotype or CIMP was applied for CRC classification based on the methylation status [168,169]. In most sporadic CRC where high MSI is observed, it is observed that patients have abnormal hMLH1 promoter methylation [170].

*Helicobacter (H.) pylori* infection has been described in CRC and has revealed to stimulate alterations in the methylation of host genes, particularly genes implicated in inflammatory pathway [171]. These genes are, for instance, *II1b*, *Nos2*, and *Tnf* [171]. Moreover, the total abnormal methylation is related with the probability of developing CRC in patients infected by *H. pylori*. Even though *H. pylori* has been identified in colorectal malignant tissues its direct influence in carcinogenesis is not elucidated [172,173]. *H. pylori* infection targets the normal gastric mucosa leading to non-atrophic gastritis [161].

An investigation indicated that infection with virulent strain of *H. pylori* that express *CagA* gene is suggested to CRC carcinogenesis through promoting IL8 synthesis [174]. The study of epigenetic as well as clinical information from The Cancer Genome Atlas (TCGA) suggested subgroups of individuals with different clinical characteristics and a group of genes as well as pathways implicated in CRC growth [175]. Furthermore, the existence of John Cunningham Virus (JC virus) in CRC has been described, and it is predicted that infection can have influence in carcinogenesis and can be implicated in the late phases of CRC progress [175,176,177,178,179]. Despite the comprehension of the epigenetic processes in single infections, the implication of epigenetic processes in microbiome-mediated CRC is not known [180].

## 8. Drug Resistance and Toxicity in Cancer Induced by Microbiome

### 8.1. Chemotherapy

**Figure 3:**
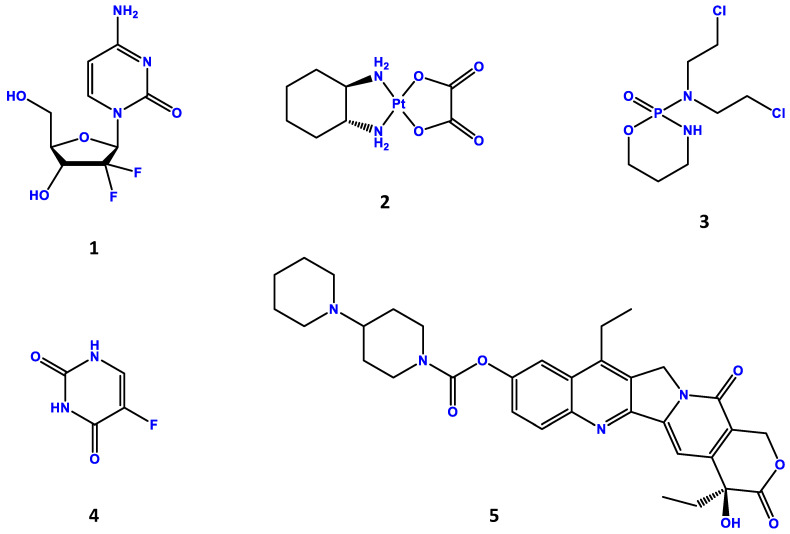
Chemical structures of gemcitabine (**1**), oxaliplatin (**2**), cyclophosphamide (**3**), 5-fluorouracil (**4**) and irinotecan (**5**).

Commensal microbes may modulate chemotherapy efficacy. For instance, *E. coli* can modulate the efficiency of two anticancer treatments, gemcitabine (**1**, Figure 3) and also CB1954, through promoting resistance as well as activating cytotoxicity in tumors, correspondingly. Gemcitabine has revealed to be metabolized by bacteria existent in human PDAC (pancreatic ductal adenocarcinoma), an influence associated with intratumoral LPS (lipopolysaccharide toxin) incidence, which can be overcome with the administration of an antibiotic therapy [181].

Various anticancer drugs revealed to possibly be modulated by different bacteria in vitro [182]. In a study using mice, oxaliplatin (**2**, Figure 3) as well as cyclophosphamide (**3**, Figure 3) have reduced efficacy in inhibiting tumor development when the studies are performed on germ-free mice or mice treated with wide-spectrum antibiotics. After antibiotics-mediated commensal reduction, tumor-infiltrating myeloid cells reacted unsuccessfully to CpG-oligonucleotide tumor immunotherapy resulting in lower TNF production, or to oxaliplatin treatment with diminished formation of reactive oxygen species as well as impaired cytotoxicity [183]. Further, chemotherapy corresponding intestinal barrier damage allows intestinal commensal translocation to secondary lymphoid organs, where they promote systemic simulation of Th17-type tumor antigen-specific CTLs in mouse models [184]. Antibiotic therapy prevents that commensal intestinal translocation and corresponding T-cell polarization, thus reducing the tumoricidal activity of chemotherapy [185]. Furthermore, microbial influences on chemotherapy efficiency, chemotherapy and associated mucosal damage may affect the intestinal microbiome constitution. Even before the extensive utilization of NGS methods, culture-based procedures revealed data regarding chemotherapeutic agents similar to 5-fluorouracil (5-FU, **4**, Figure 3) that may modulate the oral but also fecal microbiome of laboratory animals with an increase in Gram-negative anaerobes [186]. These results were after extended by 16S rRNA sequencing, showing a reduction in *Eubacterium* as well as *Ruminococcus* spp. [187].

Irinotecan (**5**, Figure 3) therapy was linked to certain intestinal microbiome dysbiotic configurations and extended expression of microbial β-glucuronidases [188]. Similarly, individuals obtaining a myeloablative conditioning treatment for non-Hodgkin’s lymphoma showed an increase in Enterobacteriaceae and Enterococcaceae as well as a reduction in Ruminococcaceae, Lachnospiraceae, and *Bifidobacterium* spp. [189]; on the other hand, allogeneic hematopoietic cell transplantation (allo-HCT) as well as immune cell reconstruction have been linked to an increase in the intestinal commensals *Faecalibacterium*, *Ruminococcus*, and *Akkermansia* spp. [190]. The influences of these chemotherapy-promoted microbial modifications in affecting tumorigenesis, therapy reactions, as well as chemotherapy-promoted adverse impacts are worth of additional research [191].

#### Irinotecan and Gut Microbiome

Topoisomerase-I enzymes are ubiquitous and are essential in different DNA mechanisms that allow life maintenance such as DNA transcription, replication, and repair. The comprehension of the role of eukaryotic topoisomerase-I resulted in the identification of this enzyme as a possible target for anticancer treatment [192].

**Figure 4:**
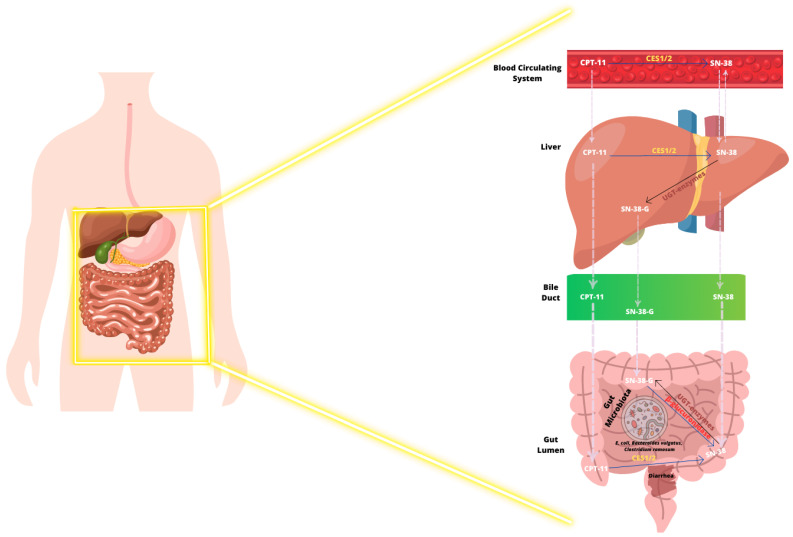
Irinotecan (CPT-11) metabolism and excretion in the human body. The deactivation and activation of compounds (CPT-11 or SN-38) are represented by dark and blue arrows, respectively. CPT-11 generated delayed diarrhea due to the toxicity promoted by bacterial species of intestinal microbiota that synthetize β-glucuronidase inducing the conversion of SN-38-G to its active metabolite structure, SN-38.

Irinotecan (CPT-11) is a camptothecin derivative that shows anticancer activity in various solid tumors. It has been extensively used as therapy for colorectal, pancreatic, as well as lung cancer [193]; further, in children, it is currently administrated essentially as a therapy for soft tissue sarcomas, bone tumors as well as neuroblastoma [194,195]. CPT-11 may be administrated alone; however, it is more commonly combined with different cytotoxic drugs (e.g., 5-fluorouracil, oxaliplatin), monoclonal antibodies (e.g., cetuximab, bevacizumab) or with kinase inhibitors [196,197]. Latest experimental as well as clinical investigations have revealed that inhibitors of DNA repair, epigenetic alterations, signaling modulators, and immunotherapy may additionally be used together with CPT-11 [198]. Irinotecan is a chemotherapeutic agent with antineoplastic activity [194], and it enters the blood circulating system as a prodrug, CPT-11, which needs enzymatic conversion by carboxylesterase (CES1 and 2) (Figure 4) [199,200]. CES1 and CES2 can be found in liver, colon, kidney, as well as blood cells; however, the conversion by these esterases predominantly takes place intrahepatically [201]. The active metabolite is SN-38 inhibits topoisomerase-I, leading to single-strand DNA breaks causing the cell cycle arrest and eventually, the cells cannot repair the accumulation of damage and initiate apoptosis [199]. Furthermore, irinotecan’s active metabolite, SN-38, is glucuronidated in the liver by UDP-glucuronosyltransferase (UGT) and it is converted in an inactive glucuronide (SN-38G), that is excreted into the gut lumen through the bile duct (Figure 4) [202].

Several symbiotic bacterial species of intestinal microbiota may synthetize β-glucuronidase that converts SN-38-G back to its active and more toxic metabolite structure, SN-38, leading to an increase in irinotecan gut toxicity. Examples of these bacteria are *Escherichia coli*, *Bacteroides vulgatus*, as well as *Clostridium ramosum* [203,204]. Wallace and collaborators [204] demonstrated that bacterial β-glucuronidases cleave the glucuronide fraction aiming to utilize it as a carbon supply, distributing the active form, SN-38, into the gut lumen, causing diarrhea (Figure 4) [204].

Bacterial β-glucuronidases (or potential candidate structures) are present in 43% of species in The Human Microbiome database. Further, the bacterial enzyme has a ‘bacterial loop’ not observed in the human form of the enzyme, allowing extremely selective inhibitors of the bacterial enzyme to be produced, two of which blocked the active site of the *E. coli* β-glucuronidase, but had no influence on bovine liver glucuronidase. The quinolone antibiotic ciprofloxacin has also been described as inhibitor of this enzyme, and small doses of amoxapine, recognized to cause inhibition of bacterial β-glucuronidases, blocked diarrhea linked to irinotecan in a rat model [205,206]. An investigation of crystal constitutions of representative β-glucuronidases from *Streptococcus agalactiae* and *Clostridium perfringens* and the Proteobacteria *Escherichia coli* as well as the Bacteroidetes *Bacteroides fragilis* has shown that these enzymes have noticeable distinctions in catalytic properties and susceptibilities for inhibition, indicating that the intestinal microbiome can guarantee functional variety in orthologous enzymes. Furthermore, minor alterations in the structure of designed inhibitors may promote major conformational alterations in the β-glucuronidase active site [203].

The use of irinotecan therapy itself may alter the host gut microbiome (GM) constitution, increasing the incidence of glucuronidases-expressing species, for example *E. coli*, *Staphylococcus* spp., as well as *Clostridium* spp. [207]. Taking into consideration the role of GM constituents on irinotecan metabolite-generated diarrhea, the possible benefit of antibiotics coadministration with irinotecan has been investigated, with positive results. The use of penicillin/streptomycin, in irinotecan-treated rats caused a decrease in the levels of SN-38 present in the feces and diminished diarrhea [208]. Despite the fact that several early investigations have pointed to the role of neomycin in decreasing irinotecan-generated delayed diarrhea [209], some later data mitigated these findings [210]. Regardless of its potential efficacy, the utilization of concomitant prophylactic antibiotics with chemotherapy is debatable, because of potential occurrence of antibiotic resistance as well as influence on GM constitution. Different approaches more precise to target-glucuronidase activity have been studied, comprising the “old” drugs, for instance Amoxapine to inhibit-glucuronidases [206]. 3D X-ray crystallographic results are also under analysis in order to logically design a glucuronidase inhibitor [204]. New pharmacological compounds have been investigated and positive effects have been observed [211]; however, their use for clinical practice has not yet been approved [212].

### 8.2. Immunotherapy

Immune-based anticancer therapies consist of a range of therapeutic methods aiming to empower the individual’s immune system or use third-party immune elements to destroy cancer cells. This method is now spearheaded by interventions targeting negative regulators of T-cell activation, named ‘‘immune checkpoints’’, which are regularly ‘‘hijacked’’ by the tumor in stimulating an immune-benefiting TME (tumor microenvironment). Checkpoint inhibitors, for example antibodies against programmed cell death protein 1 (PD-1) or its ligand PD-L1 as well as cytotoxic T lymphocyte-associated protein 4 (CTLA-4), may block the communication of T cells with their suppressive cognate ligands on tumor or stromal cells [47,213], to release an antitumor immune reaction. Consequences of this involvement, notable in a minority of individuals, differ from fully remission in sporadic occasions to substantial life extension even in metastatic cancers (metastatic melanoma, non-small-cell lung cancer, Hodgkin lymphoma, as well as renal cell carcinoma as representatives). In 2015, two mouse investigations revealed that members of the commensal intestinal microbiome such as *Bifidobacterium* spp. were able to increase the antitumor efficiency of PD-L1 checkpoint blockade [214], while *Bacteroides thetaiotaomicron* and *B. fragilis* were linked to increased CTLA-4 inhibitor effectiveness [214,215]. Moreover, the antitumor effectiveness of PD-1/L1-targeting treatments were linked to various bacteria, such as *Akkermansia*, *Faecalibacterium*, *Clostridiales*, as well as *Bifidobacterium* spp. [216,217,218].

Increased levels of fecal SCFA (short-chain fatty acids) have been linked to extended progression-free survival or improved antitumor reactions, but increased systemic levels were linked to worse therapy reactions [219]. Butyrate can also restrict the ability of dendritic cells to stimulate tumor-specific T cells and memory T cells, thus limiting the effectiveness of anti-CTLA-4 ICI (immune checkpoint inhibitor) [220]. Another microbial metabolites also influence ICI. For instance, *Bifidobacterium pseudolongum*-produced inosine improves ICIs by the activation of A_2A_ receptors on T cells [221]. Other ways of microbe-host relations in cancer immunotherapy involve direct induction of dendritic cells in lymph nodes by *Akkermansia muciniphila* to enhance the antitumor efficiency of ICIs in an IL-12-dependent way [218] or by *Bacteroides* spp. by stimulation of Th1 and CD8+ T-cell antitumor immune reactions [215,218].

#### 8.2.1. Immunotherapy in Advanced Melanoma and the Gut Microbiome

The development of therapies targeting immune checkpoints, for example programmed death-ligand 1 (PD-L1), programmed cell death protein 1 (PD-1) as well as cytotoxic T-lymphocyte-associated protein 4 (CTLA-4) with immune checkpoint inhibitors (ICIs) has extraordinarily changed the course of melanoma treatment, currently making possible to fight advanced melanoma with remarkably higher success. Various landmark randomized controlled trials have demonstrated significant and durable survival benefits, causing alterations to standard of care internationally [222,223]. Currently, over 50% of patients that received an administration of combined PD-1 and CTLA-4 blockade are alive after five years. Regardless of these progresses, less than half of the patients that had an administration of a single-agent ICI respond to it, while a higher response to a combination of PD-1 and CTLA-4 is linked to recurrent toxicity with immune-associated adverse effects [224,225].

The study of an association between the intestinal microbiome and reaction to ICIs, in melanoma as well as other tumors, demonstrated that the intestinal microbiome has potential as a biomarker of reaction to therapy [216,217] in addition to a therapeutic target [226,227]. Even though there is substantial data for particular gut microbial characteristics linked to positive responses in mouse investigations [215,228], occurs a major disagreement on which microbiome features are related to therapy reactions in the human setting (Table 1). In one of the largest metagenomic investigations so far, Routy et al. [218] discovered responders to harbor substantially increased relative incidence of *Akkermansia muciniphila*, *Alistipes* and in general more *Firmicutes* compared to non-responders [218], while Gopalakrishnan et al. [216] discovered an increased relative incidence of *Faecalibacterium prausnitzii* in responders compared with non-responders (Table 1). Additionally, Matson et al. [217] discovered that responsiveness to PD-1 treatment was characterized by a higher relative incidence of a group of eight species driven by *Bifidobacterium longum*. Frankel et al. [229] described that microbiota changed by ICI regimen; however, the higher incidence of *Bacteroides caccae* was frequent in responders treated with any ICI regimen (Table 1) [229]. Various confounding elements can play a role in this lack of agreement, for instance collection and DNA extraction protocols, dietary and variations in drug use across countries, problems of specimen size and statistical power, variability in microbiome signatures between responders as well as functionally associated microbial signals, yet intrinsic to every cohort. Cohort has influences ranging from population-specific features to methodological choices in specimen processing as well as laboratory analysis, which are main issues in microbiome investigations [230,231]. Therefore, larger and varied cohorts with metagenomic data as well as standardized metadata are required to improve the elucidation of the microbiome determinants of reaction to immunotherapy [232].

Anti-programmed cell death protein 1 (anti-PD-1) treatment is responsible for long-term clinical improvements in patients diagnosed with advanced melanoma; further, the constitution of the intestinal microbiota is associated with anti-PD-1 efficiency in preclinical models and cancer patients. A clinical trial designed to study whether anti-PD-1 resistance may be surmounted by altering the intestinal microbiota, analyzed the safety and efficiency of responder-derived fecal microbiota transplantation (FMT) along with anti-PD-1 in patients with PD-1-refractory melanoma. This combination was well tolerated, has shown clinical improvements in 6 of 15 patients, furthermore responders revealed higher incidence of taxa that were previously demonstrated to be related to a response to anti-PD-1, higher CD8+ T-cell activation, as well as decreased incidence of interleukin-8 expressing myeloid cells. This investigation has shown that FMT and anti-PD-1 altered the intestinal microbiome and reprogrammed the tumor microenvironment to surmount resistance to anti-PD-1 in a subgroup of PD-1 advanced melanoma [233]. So, the more we understand about the impact that immunotherapies have on the gut microbiome, the more we will be able to use it to overcome resistance to different immunotherapeutic agents and this will definitely represent a step forward in cancer treatment.

#### 8.2.2. Microbiome and CAR-T Therapy

Chimeric antigen receptor (CAR) T-cells are autologous T-cells re-directed towards a tumor-specific antigen. They are progressively being studied in various tumor types that are relapsed/refractory in addition to frontline disease settings, mainly in hematologic malignancies (HM) because it revealed to be an efficient approach for patients with refractory B-cell hematological malignancies. CAR T-cells, now authorized in HM treatment, are linked to harmful impacts, for instance cytokine release syndrome (CRS), neurotoxicity, as well as suppression of humoral immunity caused by B-cell aplasia [234,235,236]. As we mentioned before, more and more data indicate that the microbiome can modulate the efficiency of cancer immunotherapy. In a B cell lymphoma patient cohort, it was demonstrated that large-spectrum antibiotics therapy (‘high-risk antibiotics’) before CD19-targeted chimeric antigen receptor (CAR) T-cell treatment is linked to harmful effects. Considerable associations concerning pre-CAR-T infusion *Bifidobacterium longum* and microbiome-encoded peptidoglycan biosynthesis, as well as CAR-T therapy-related 6-month survival or lymphoma evolution, were observed [237]. In a different investigation, they studied the role of the gut microbiome on adverse effects in multicenter research of patients with B cell lymphoma and leukemia. They determined that alterations in the gut microbiome are linked to clinical effects after anti-CD19 CAR T-cell treatment in patients with B cell malignancies [238].

## 9. Allogeneic Hematopoietic Stem Cell Transplantation

Allogeneic hematopoietic stem cell transplantation (allo-HSCT) combines stem cell treatment, conventional treatment (with chemotherapy, radiation or antibodies) in addition to immunotherapy [239]. Allo-HSCT is based on a conditioning regimen, which includes chemotherapeutic agents producing or not whole-body irradiation and/or antibodies, that eliminate cancer cells and make it possible for the recipient immune system to be able to then receive an immune system-rebuilding combination of donor HSCs. Additionally, allogeneic donor T cells may attack residual tumor cells, leading to graft-versus-tumor (GVT) activity. Nevertheless, these alloreactive donor T cells may also attack target organs of the host, which includes the skin, liver, intestine, thymus, central nervous system, ovary or testis in addition to the haematopoietic system (named graft-versus-host disease (GVHD)) [239,240,241,242,243].

The intestinal microbiota represents a powerful modulator of systemic immune responses. Further, there is increasing data supporting that microbiota may remarkably influence cancer immunosurveillance [181,214,215,244,245,246,247]. Different investigations revealed that gut microbiota may affect the immune response to systemic cancer chemotherapy, radiotherapy as well as immunotherapy [248,249]; further, disruption of the intestinal microbiota is linked to resistance to cancer treatment [181,183]. GVHD (particularly chronic GVHD) is inversely linked to relapse [239,240,250] as the allogeneic T cells leading to GVHD also promote GVT activity. Since the development of GVHD is linked to alterations in the gut flora as we mentioned before, investigations to study modifications in the gut microbiota and relapse after allo-HSCT seem justified. A retrospective observational investigation of 541 patients undergoing allo-HSCT at a single centre recognized a cluster of bacteria mostly constituted by *Eubacterium limosum* that might work as a biomarker of relapse risk: increased incidence of this cluster was linked to reduced relapse [249]. Quantitative species-based measures, for instance inverse Simpson index and Shannon diversity index were frequently utilized to summarize and compare the microbiome alpha variety in distinct communities [251]. To investigate the possible influence of the gut microbiota on allo-HSCT complications, Taur et al. investigated allo-HSCT recipients from a particular institution, analyzing fecal specimens collected at the time of neutrophil recovery [252] and discovered that an increased gut microbiome variety was considerably linked to an increased generally survival as well as lower transplant-associated mortality compared to patients with reduced variety (Inverse Simpson less than 2). An additional investigation showed that a higher gut bacterial variety was specifically linked to lower mortality from GVHD, while correlations between variety and malignant relapse were not observed [253]. Remarkably important, the elucidation of a correlation between an increased variety of the gut microbiota and a reduced risk of transplant-associated mortality was afterwards confirmed in an investigation with large multicenter international cohorts [254]. However, considering the fact that the microbiome is varied even geographically, it is critical to have multiple investigations regarding the alterations that occur in the microbiome when allo-HSCT and GVHD occurs, so that beforehand can be standardized for clinical use the prediction of complications and disease relapse.

### 9.1. Fecal Microbiota Transplantation as a Preventive Approach in Allo-HSCT

As we mentioned above, the disruption of intestinal microbiota has been associated with major problems in allogeneic hematopoietic stem cell transplantation (allo-HSCT) recipients. Therefore, different approaches have been suggested to decrease dysbiosis as well as associated complications [255].

Fecal microbiota transplantation (FMT) is a promising and possibly helpful approach in allo-HSCT recipients [255]. FMT is based on the combination of fecal substances from a healthy donor into the gastrointestinal tract of a recipient carrying a disrupted intestinal microbiome. The origin of the fecal substances can be autologous, with feces collected prior to the onset of dysbiosis, or from a related or unrelated healthy donor. Due to having a similar genetic background as well as similar environment, a related FMT donor may have a similar GM constitution, which may be not recommended in several cases [256]. Because of its possible capacity to re-establish an eubiotic intestinal microbiome layout in the recipient, FMT has been suggested as a therapy of other clinical illnesses, such as inflammatory bowel disease, with encouraging primary results [257].

Even though there is an emerging possible clinical benefit of FMT in allo-HSCT patients, the risk of infections caused by the transport of living microbial consortia to an immunocompromised host with compromised intestinal permeability must be the highest concern [258]. Therefore, supplementary information on the biological mechanisms behind clinical results is required, so that the use FMT is possible in clinical practice; further, the safety profile as well as efficiency of the procedure must be verified to improve the understanding of the role of FMT in allo-HSCT recipients [255].

### 9.2. Colonization Screening to Guide Antibiotic Therapy in Allo-HSCT

An investigation conducted by Dhanya et al. analyzed the clinical importance of colonization screening cultures to monitor empirical antibiotic treatment in allo-HSCT [259]. This cohort of patients predominantly included recipients of HSCT for hemoglobinopathies, in whom previous exposure to chemotherapy as well as intravenous (IV) antibiotics was not usual. Therefore, these results might not be generalizable to patients that were submitted to transplantation for hematological malignancies. Screening of colonization by resistant strains is a possibly helpful approach, as part of structured stewardship programs, and this is verified by different data regarding the influence of multidrug-resistant (MDR) bacterial colonization on transplantation consequences [260,261]. These moderately conflicting conclusions emphasize the necessity for additional investigations in order to better comprehend the clinical importance of colonization monitoring [262].

## 10. Conclusions

Cancer is an increasingly complex disease each time we try to understand it at a deeper level; in this article, we summarize recently discovered cancer hallmarks. Although the microbiome is an important part of the human body due to its varied functions, most investigations reveal that, far more than expected, it plays a fundamental role in carcinogenesis. The gut microbiome is the most studied because it has key functions in the human body beyond most microbiomes inhabiting the human intestine; however, the human microbiome is varied, largely unknown and it has a lot of interesting particularities that are not completely elucidated.

The genetic field has revealed interesting discoveries because of the development of new sequencing tools as well as bioinformatic technology, which can ultimately provide a better understanding of multiple diseases such as cancer; here, we have correlated cancer with genetic and epigenetics changes induced by the microbiome, and we found that it is a topic that requires more investigation. Apart from that, it was possible to elucidate that the microbiome, directly or indirectly (toxins), can not only damage DNA but also provide resistance to different anticancer treatments.

For future investigations, it is essential to have multidisciplinary teams to study anticancer resistance acquired by an altered human microbiome in patients that does not respond to the conventional therapies in various cancer types, so that possible alternatives to fight this disease can be developed.

## Figures and Tables

**Table 1:** Microbiome features acquired by immunotherapy reactions.

Authors	Microbiome Features
Routy et al. [221]	↑ *Akkermansia muciniphila*, *Alistipes* and *Firmicutes*
Gopalakrishnan et al. [219]	↑ *Faecalibacterium prausnitzii*
Matson et al. [220]	↑ Group of eight species driven by *Bifidobacterium longum*
Frankel et al. [232]	↑ *Bacteroides caccae*

## Data Availability

Not applicable.
